# Teachers’ Implementation of Inclusive Teaching Practices as a Potential Predictor for Students’ Perception of Academic, Social and Emotional Inclusion

**DOI:** 10.3389/fpsyg.2022.917676

**Published:** 2022-07-28

**Authors:** Ghaleb H. Alnahdi, Katharina-Theresa Lindner, Susanne Schwab

**Affiliations:** ^1^Department of Special Education, College of Education, Prince Sattam Bin Abdulaziz University, Al-Kharj, Saudi Arabia; ^2^Center for Teacher Education, University of Vienna, Vienna, Austria; ^3^Research Group Optentia, North-West University, Vanderbijlpark, South Africa

**Keywords:** inclusive education, SEN, inclusive teaching practices, Saudi Arabia, inclusion

## Abstract

The aim of the study was to illustrate the impact of teachers’ implementation of differentiation and individualization (perceived by students) on students’ perception of their inclusion regarding their social inclusion, emotional wellbeing and academic self-concept. The study sample comprised 824 third-to-eighth-grade students [255 males (31%) and 569 females (69%)]. Around 10% of the sample (82) had special educational needs (SEN). Students’ perceived inclusion levels and academic self-concept were examined with the Arabic version of the Perceptions of Inclusion Questionnaire (PIQ-S-AR). Students’ ratings of inclusive practices in their classroom were examined using the Arabic version of the Inclusive Teaching Practices Scale (ITPS). SEN students expressed lower perceived social inclusion, emotional inclusion, and academic self-concept in comparison with non-SEN students. Moreover, high levels of inclusive teaching practices strongly predicted students’ perceived emotional inclusion, social inclusion, and academic self-concept. The results of the study supported the importance of school-level inclusive teaching practices and their relation to students’ school experiences. It also highlighted the need for schools and teachers to work towards improved school-level inclusion experiences for SEN students.

## Introduction

In Saudi Arabia’s recently changed approach to inclusive education, there has been clear progress in access to education for all. Over the past two decades, access to regular school settings for students with disabilities has been established throughout the country. It is now possible for students with learning disabilities to join regular classrooms, and students with other disabilities are now accommodated in regular schools, although they attend separate classes (Al-Mousa, 2010; Alnahdi et al., 2019). Nevertheless, the provision of access to regular schools with separate classes does not equate with inclusion, and the question must be asked as to whether students with and without special educational needs (SEN) are being integrated enough to create an inclusive school environment and whether inclusive education practices are applied in the classroom.

When applying inclusive education practices, Saudi Arabian teachers face severe challenges in terms of material and human resources (Maghrabi, 2013; Almalki and Abaoud, 2015; Housawi and Ibn Rageh, 2015; Alrayss and Algmeay, 2016; Alssissi, 2017). For instance, classes are overcrowded and there are not enough teachers and other pedagogical staff, which in turn leads to a lack of opportunities for team teaching which could provide better support for students in inclusive educational settings (Alnahdi, 2014). There has been little research on students’ perspectives and perceptions of educational inclusion in the Saudi Arabian context. International studies on students’ perception of inclusive classroom processes regarding teaching and learning show that individual students of one class who are receiving the same instructional practices perceived their teachers actions differently (Göllner et al., 2018). In example, previous studies highlighted students’ gender as predictor for their perception of learning and teaching processes initiated by teachers revealing that female students perceived higher levels of differentiation and personalization than their male peers (Tennant et al., 2015; Lindner et al., 2019). In the context of processes on peer level, Dare et al. (2017) looked at acceptance of the behaviour of fellow students with learning difficulties including peer relationships and inclusive behaviour during play and found that boys perceived a higher level of social acceptance with peers with learning difficulties. Regarding SEN as potential predictor of students’ perceived level of inclusion, international studies showed lower social inclusion scores (e.g., operationalized through social support or frequency of having friendships in class) for students with SEN compared to their peers without SEN (Schwab et al., 2013, 2021). Considering the knowledge gap in inclusive education in Saudi Arabia, research on a possible link between the perception of inclusive teaching practice from the students’ perspective and perception of their own inclusion in class is needed. The study considers students’ views on academic, social, and emotional inclusion in connection with their perception of teachers’ actual implementation of inclusive practices in school settings in Saudi Arabia. It seeks to inspire further innovation in examining the implementation of inclusion with a focus on students’ perspectives as a further step in establishing an approach to education for all.

### Inclusive Teaching Practices for Equity in Education

Due to the trend towards more inclusive education, teachers are facing new challenges in the adequate implementation of teaching methods. From the teachers’ perspective, using inclusive teaching practices such as differentiation (regarding groups of students) and personalization (regarding individual students) are useful for working with a diversity of students (Lindner and Schwab, 2020; Schwab, 2021). Tomlinson (2014) describes differentiated instruction as a necessary teaching approach for a heterogeneous student population. A fruitful learning environment for every student (with and without special educational needs) can be provided by setting individual academic goals, ongoing external and self-assessment, flexible tasking and grouping strategies, and respect for the individual characteristics of all the students in a class (Tomlinson, 2014). The shift from segregated schools for students with and without SEN has been accompanied by research focusing on common schooling for both groups of students and the efficacy of common schooling for students with SEN (e.g., Ruijs and Peetsma, 2009). Due to the diverse characteristics of students, the enablement of participation therefore strongly depends on the design of pedagogical offers and teaching approaches (McMurray and Thompson, 2016; Petersen, 2016; Ainscow and Messiou, 2018). Against this background, making inclusion of all students happen is considered to be the objective of teachers’ implementation of inclusive teaching practices. In this context, it seems necessary to investigate, whether this demand—providing students inclusion through implementing inclusive teaching practices—can be fulfilled from the perspective of the measures’ recipients, namely the students themselves. Within this research area, studies on the relationship between inclusive teaching practices and students’ perceptions of inclusion on school- and classroom-levels regarding formal (explicit learning and teaching processes) and informal (social interactions in general, play) educational processes are rare. Moreover, uncertainty remains as to whether students’ perceptions of inclusion, which can be operationalized through their academic self-concept, school wellbeing, and social participation (Venetz et al., 2015), can be considered outcome variables of inclusive teaching approaches.

### Outcomes of Inclusive Education

When dealing with inclusive educational settings (such as classrooms and schools), there is a question of how school-level inclusion outcomes can be operationalized. Of course, students’ academic achievements are outcome factors of teaching and learning (Gore et al., 2021; Johnson et al., 2021). Therefore, students’ performances in specific curriculum-related domains could be assessed to check whether inclusive teaching approaches such as differentiated instruction, are successful considering outcome-oriented evaluation (Deunk et al., 2018). The literature regarding SEN students’ academic achievements has shown that compared to when they are segregated into special classes or schools, they achieve better academic performances when they are allowed to integrate into regular classrooms (Krämer et al., 2021). The academic performances of non-SEN students in inclusive classes are at the least similar to the academic outcomes of non-SEN students in regular classes (Ruijs and Peetsma, 2009; Ruijs et al., 2010; Dessemontet and Bless, 2013; Dell’Anna et al., 2021). However, measuring students’ academic achievements is not the only way to operationalize the impact of inclusive education on students. As Schwab (2021) pointed out, alongside academic achievement, post-school options, effective inclusive teaching practices, and students’ socio-emotional development should also be considered. In particular, students’ social inclusion, their school wellbeing, and their academic self-concept (see Venetz et al., 2019) should be addressed within the evaluation framework of inclusive education.

### Academic Performance Versus Academic Self-Concept

Several researchers have stressed that students’ academic self-concept plays a major role in the context of inclusive education (Elbaum and Vaughn, 2003; Stiefel et al., 2018). Cambra and Silvestre (2003, 201) defined academic self-concept as a conglomerate of “students” assessment of their own learning abilities, academic performance, and relationships with teaching staff’. Regardless of actual academic achievement, by focusing on students’ academic self-concept, students with different levels of achievement can be compared and thus, the effectiveness of inclusive teaching practices can be operationalized (Vaughn et al., 1996). Kocaj et al. (2018) investigated the relationship between educational school placement and SEN students’ academic self-concept and found that SEN students reported a more positive academic self-concept when schooled in special educational schools compared to their SEN peers who attended regular classes. Providing a possible reason for this finding, Kocaj et al. (2018) allude that these students gained the possibility of comparing performances with peers who were at the same academic level; they suggested that this was a positive predictor for the students’ academic self-concept. Within the framework of their study, Roy et al. (2015) highlighted that the use of differentiated and personalized instruction as part of inclusive teaching practices has a positive effect on students’ academic self-concept; moreover, it lessens the negative effect of the students’ average achievement in class, which can adversely affect their academic self-concept (also known as the big-fish-little-pond concept; Fang et al., 2018). By applying hierarchical linear modelling to a sample of 422 elementary students, Roy et al. (2015) showed that differentiated instruction could indeed lessen the negative effect of big-fish-little-pond on students’ academic self-concept, especially in the case of students with low individual academic achievement. As a possible explanation for this finding, they describe differentiated instruction (Tomlinson, 2014) as a strategy for student encouragement that could help students develop self-assessments regarding their academic performance, instead of comparing their academic achievements to that of their peers (Roy et al., 2015). The study by Roy et al. (2015) did not consider SEN as a possible predictor for the effect of students’ average academic performance on their academic self-concept, but especially in the context of inclusive education, the examination of SEN as a possible predictor is of interest.

### Students’ School Wellbeing as an Outcome Variable of Inclusion

Focusing on the cognitive output of students as well as their non-cognitive aspects is another way to operationalize the effectiveness of inclusive teaching and learning. Therefore, students’ wellbeing or degree of emotional inclusion in school is often used as an outcome variable for teaching practices. Engels et al. (2004, p. 128) described students’ wellbeing in school as “a positive emotional state that is the result of a harmony between the sum of specific context factors on the one hand and the personal needs and expectations towards the school on the other hand.” This definition of school wellbeing assumes that, to feel a certain sense of wellbeing as a student, one should be able to adjust to the school’s expectations and demands. Likewise, the school itself must make every effort to meet the needs of its students (Tomé et al., 2021). In their quantitative study, Van Petegem et al. (2008) investigated students’ characteristics, the motivational aspects of students’ school attendance, and students’ perception of teacher behaviour as possible predictors for students’ school wellbeing. They observed that students who reported a stronger desire to attend school and learn had a stronger sense of school wellbeing. They also investigated personal identification with the learning content as another predictor of students’ wellbeing (Van Petegem et al., 2008). In the context of inclusive education, this seems particularly relevant, since inclusive teaching methods facilitate the differentiation and personalization of learning content, regardless of the level of achievement. Therefore, the question arises as to whether inclusive teaching practices such as differentiation and personalization enhance the perception of wellbeing in school of SEN students.

### Social Participation in Inclusive Classrooms

Students’ social participation is often considered to be the most important argument for why SEN students should be included in regular classes (see Schwab, 2018). Furthermore, students’ social inclusion can also be considered as an outcome factor for inclusion in school. Koster et al. (2009) defined SEN students’ social participation in regular education as the existence of “positive contact/interaction” between SEN students and their peers. They further described it as “the acceptance of them by their classmates,” “social relationships/friendships between them and their classmates,” and as students’ perception that “they are accepted by their classmates” (Koster et al., 2009, p. 135). Within a social network study framework, Avramidis (2013) was able to refute the notion of SEN students feeling socially excluded in class and showed that they felt equally accepted compared to the non-SEN students (although students with SEN had fewer friends than their peers without SEN). Additionally, Avramidis (2013) observed that the SEN students in regular schools saw their academic achievements in a positive light and reported a strong positive academic self-concept because they received adequate support in relation to their individual learning goals and needs (Avramidis, 2013). In another study, Avramidis et al. (2018) focused on the four key themes of social participation stated by Koster et al. (2009), namely contacts/interactions, acceptance by classmates, social friendships/relationships, and their perception of acceptance. They reported that SEN students are alone more often and that they tend to have fewer friendships with classmates in comparison with their peers without SEN. A possible explanation for the authors’ findings is that there may be a lack of adequate teaching approaches for engaging SEN and non-SEN students in interactions with each other; moreover, the use of inappropriate instructional methods that unintentionally discourage SEN students from engaging in valuable social interactions with their non-SEN counterparts may also play a role. Taking into consideration the key factor of social self-perception, SEN students reported a more positive self-perception of their acceptance and inclusion than their actual reported relationships in class could justify (Avramidis et al., 2018). Within the scope of a phenomenological approach, Dare et al. (2017) dealt with the question of how Saudi Arabian students perceived social inclusion and exclusion of their peers with learning difficulties. Results show that students reported more behavioural examples encompassing inclusive characteristics that mostly concerned friendships, inclusive play, and support of studying. Against this background, it is relevant to consider the students’ perceptions of teachers’ use of inclusive teaching practice as an extra variable to investigate regarding its influence on students’ perception of social inclusion. Does the perceived inclusive behavioural of the teacher influence the perception of one’s own social inclusion?

### The Perception of Inclusion

One method to assess the possible outcomes of inclusive teaching practices, namely the students’ perception of inclusion in class, is the use of the Perceptions of Inclusion Questionnaire (PIQ; Venetz et al., 2015). The questionnaire is available free of charge in over 20 different languages (e.g., German, Arabic, Afrikaans, French, Italian, and Spanish) and allows researchers to gain insights into students’ emotional inclusion, social inclusion, and academic self-concept. The use of the instrument has shown the importance of giving students a voice, thus shedding light on their different perspectives. While Venetz et al. (2019) used the PIQ to compare students’ self-reported inclusion in school to teachers’ ratings, Schwab et al. (2020) compared studnnts’ self-reports with teacher and parents’ reports. The results of Schwab (2021) showed that teachers and parents systematically rated the social inclusion and the academic self-concept of SEN students lower than the students themselves did. Regarding the key factor of school wellbeing, only teachers rated the emotional inclusion of SEN students more negatively than the students themselves did. A possible explanation for these rating variances between the different sample groups could be the perception of SEN students as students who are less accepted and integrated. However, the consistency between the students’ self-reports and reports from parents and teachers to the students’ academic self-concept showed that the external ratings (provided by teachers and parents) were more strongly related with the actual academic achievements of the students (Zurbriggen et al., 2019). Against such a background, it seems necessary to consider not only teachers’ and parents’ perspectives, but also the self-reports of the students themselves in their assessments of school-level inclusion. Regarding the perception of students, results of the studies using the student version of the PIQ show lower levels of academic self-concept (DeVries et al., 2018; Zurbriggen et al., 2018; Alnahdi and Schwab, 2020; Guillemot and Hessels, 2021) and/or emotional inclusion (DeVries et al., 2018) for students with SEN than their peers without SEN.

### The Present Study

The current study aimed to investigate students’ perception of social participation, school wellbeing (emotional inclusion) levels, and their academic self-concept. In addition, it also examined the influence of the implementation of inclusive teaching practices, namely differentiation and personalization, on students’ perceptions of inclusion. Against the background of theoretical framework of the study, the goal of inclusive teaching is not only to give students with SEN adequate access to learning situations and to promote their learning processes, but also to support the inclusion of all students in class regardless of whether or not having an existing diagnosis of SEN on social, pedagogical, and academic levels (Wocken, 2014). The aim of this study is to illustrate the impact of teachers’ implementation of differentiation and individualization (perceived by students) on students’ perception of their inclusion regarding their social inclusion, emotional wellbeing and academic self-concept following the PIQ (Venetz and Zurbriggen, 2015).

Since the psychometric properties of the Arabic version of the PIQ as a scale (that includes three latent variables) was examined for the first time, an evaluation of the measurement instrument with regard to its psychometric quality criteria follows before the hypotheses are tested.

In the context of previously explicated research desideratum and against the theoretical framework of the study, the following research questions and hypotheses were formulated:

1. Are there group differences based on students’ characteristics (having SEN, and students’ gender) in students’ ratings of their perception of inclusion?

*Hypothesis 1a*: It is expected that students with SEN will perceive lower social inclusion levels and weaker academic self-concept levels compared to their peers without SEN.

*Hypothesis 1b*: It is expected that students with and without SEN differ in their perception of school wellbeing in so far as students with SEN perceive lower levels of school wellbeing.

*Hypothesis 1c*: It is expected students’ perception of inclusion might differ by students gender.

2. Do students’ perception of inclusive teaching practices predict students’ perception of inclusion regarding their social inclusion, academic self-concept and school wellbeing?

*Hypothesis 2*: It is expected that students who perceive higher levels of inclusive teaching practices perceive themselves as more included regarding their social inclusion, academic self-concept and school wellbeing than students who perceive lower levels of their teachers’ implementation of inclusive teaching practices. Material and methods.

## Materials and Methods

The current study’s sample comprised 21 inclusive classes from seven elementary and two middle schools in the Riyadh region in the centre of Saudi Arabia. These schools from different areas in the Riyadh region, to collect data from more representative sample. However, schools with collaborating teacher willing to distribute and collect surveys were considered in choosing schools. The definition of the inclusive class concept holds that there is at least one SEN student in the class. Students with SEN have the right to special education services owing to the fact that they have been diagnosed with learning disabilities. The special education service encompasses individualized support in the form of an additional special education teacher who conducts several lessons per week. Overall, 824 third-to eighth-grade students participated in the study [255 male students (31%) and 569 female students (69%)]. About 10% of the sample (*n* = 82) were SEN students (56 females and 26 males). Around 30% were 8 and 9 years old, 29% were 10 and 11 years old, and the rest were from 12 to 15 years old. Questionnaires were distributed as a paper version by assistant researchers to teachers in the included schools. The assistant researchers also provided the instructions and clarifications to students, when it was needed, during responding to the items. The questionnaires were completed during school time.

### Measures

#### The Perception of Inclusion Questionnaire

To assess students’ emotional inclusion (e.g., “School is fun”), social inclusion (e.g., “I have a lot of friends in my class”), and academic self-concept (e.g., “I do well in my schoolwork”), students were asked to fill out the student version of the PIQ (Venetz et al., 2015). The questionnaire’s three subscales consist of four items rated on a four-point Likert scale (1 = Not at all true; 2 = Somewhat not true; 3 = Somewhat true; 4 = Certainly true). High reliability (0.80 ≤ *α* ≤ 0.91) was shown, and the three-dimensional factor structure was confirmed using confirmatory factor analysis. Zurbriggen et al. (2017) confirmed the scales’ three-factor structure, as all items behaved normally. The use of the PIQ is extended for samples of students from grade 3 to grade 9 (Venetz et al., 2019). As the instrument had never been used in Saudi Arabia before, this study aimed to analyze its psychometric properties.

#### The Inclusive Teaching Practice Scale

To assess whether the teachers’ use of inclusive teaching practices was related to students’ social participation, academic self-concept, and emotional inclusion, this study utilized the ITPS (Schwab et al., 2022). To investigate inclusive instructional approaches as predictors for students’ inclusion perceptions, actual inclusive teaching practices from the perspective of students were examined. The ITPS consists of 14 items and can be categorized into two subscales: differentiation (e.g., “During the lesson, my teacher uses a variety of grouping strategies”) and personalization (e.g., “During the lesson, my teacher considers my interests”). Each of the subscales has seven items. The students were asked to rate these items on a four-point Likert scale (1 = Not at all true; 2 = Somewhat not true; 3 = Somewhat true; 4 = Certainly true). The psychometric properties of the research instrument regarding samples from fourth-grade students, secondary students, and teachers had already been confirmed. For the sample consisting of fourth-grade students, Cronbach’s alpha ranged from 0.77 to 0.86; thus, the internal consistency for both the subscales was provided. For this sample, the two-dimensional data structure was confirmed with the CFA (Lindner et al., 2019). For the sample of secondary students, Cronbach’s alpha was 0.81, and the internal consistency for both subscales was satisfactory. Further, the two-dimensional factorial structure was also confirmed (Schwab et al., 2022).

#### Translation

As already stated at the beginning, the scales used in the present study were not previously used in the Arabic language context. Therefore, the first step was to translate the items in a scientifically validated manner. The processes of translating scales for this study, including the back-translation technique, were carried out in accordance with the Guidelines for the Process of Cross-Cultural Adaption of Self-Report Measures (Beaton et al., 2000). First, the independent translations by two special education experts resulted in two different Arabic versions of the instruments. Through mutual agreement, these differing versions were discussed and combined into one version. The elaborated Arabic version was then presented to a bilingual (Arabic-English) expert in English language to translate the newly created Arabic version into English. After this process, the new English questionnaires were compared to the original ones to ensure that the context and the wording matched. For verification purposes, the scales were used in a pilot study with a sample of 53 elementary students. The students in the sample showed good internal consistency for both PIQ and ITPS (see Table 1).

**Table 1:** Reliability statistics for Perceptions of Inclusion Questionnaire (PIQ) subscales and Inclusive Teaching Practices Scale (ITPS).

	Alpha (all sample)	Alpha (pilot *N* = 53)	Items
EI	0.633 (0.713^*^)	0.663 (0.780^*^)	4 (3^*^)
SI	0.517 (0.605^*^)	0.666 (0.757^*^)	4 (3^*^)
AC	0.432 (0.548^*^)	0.481 (0.692^*^)	4 (3^*^)
ITPS	0.877	0.870	14

* Value after removing the negatively phrased item.

EI, emotional inclusion; SI, social inclusion; and AC, academic self-concept.

### Ethics

Participation in the study was voluntary. Approval for this study was obtained from the institutional review board (IRB) of the university where the study was carried out. The school administrations obtained the consent of authorized family members. Lastly, all the participants’ parents gave their consent for the collection and processing of data.

### The Psychometric Properties of the PIQ

Two statistical analyses were conducted to examine the reliability and validity of each scale used in this study. The construct validity of the PIQ-AR was examined through a confirmatory factor analysis. The fit of the data showed poor fit to the three-factor structures, with RMSEA = 0.084, CFI = 0.805, and x2 to degree of freedom ratio = 5.77; furthermore, CMIN = 294.510 (51) *p* < 0.05 and GFI = 0.925. The errors of items 4, 8, and 12 were covariate together, as this was recommended by the AMOS software. This makes some sense in the context, as these were the only three items that were negatively phrased. By carrying out this change, the model was improved, and RMSEA = 0.049 indicated a good fit of the model (Hu and Bentler, 1999; Schermelleh-Engel et al., 2003). In addition, the comparative fit index (CFI) was found to be 0.938, within the acceptable range (Pugesek et al., 2003), the chi-square to degree of freedom ratio was >3 (= 2.61; Kline, 1994), and GFI was found to be 0.968, indicating a good fit (Schermelleh-Engel et al., 2003). Only the chi-square value was found to be significant, with CMIN = 125.580 (48) *p* < 0.05; this was expected because of the large sample size, which occurred even in cases where the data did fit the model (Byrne, 2010). However, the reliability statistics were not supportive in that direction, with Cronbach’s alpha for the three subscales measuring below 0.7. It was found that, as the Cronbach’s alpha was similar to CFA, the three negatively phrased items were perceived differently by the students. After removing these three items—one item from each subscale—the reliability alphas improved slightly; however, 0.7 for two of the three subscales was not attained (Table 1). Therefore, a decision was made to continue all further analyses without these three items (4, 8, 12), but to continue with three items in each of the subscales: emotional inclusion (EI; 0.713), social inclusion (SI; 0.605), and academic self-concept (AC; 0.548). Please see Table 1 for more details.

With regard to the Arabic version of the ITPS scale, the construct validity was examined through CFA to ensure that the observed data fit the hypothesized two-factor scale. The results indicated that the data did fit the model with good indices (Lindner and Schwab, 2020): CFI (0.954), RMSEA (0.049), x2 to degree of freedom ratio < 3 (2.62), and CMIN = 188.891 (72) *p* < 0.05. In sum, the Arabic versions of both the PIQ and the ITPS showed acceptable psychometric properties and were considered to be ready for use. Cronbach’s alpha was computed to examine the internal consistency of the ITPS, and it reflected a good range (α = 0.887; George and Mallery, 2003).

## Results

To answer the research questions, the data were analyzed with different statistical tests. Analysis of the variance (ANOVA) was conducted to examine SEN students’ perceived emotional inclusion, social inclusion, and academic self-concept in comparison with other students. Multiple regression analyses were conducted to find out which independent variables would predict students’ perceived levels of emotional inclusion and social inclusion and their academic self-concept (based on the mean values for each of the three subscales).

### SEN in All Three Subscales

Analysis of the variance (ANOVA) was conducted to examine SEN students’ perceived emotional inclusion, social inclusion, and academic self-concept in comparison with other students. Table 1 shows that in comparison with other students, SEN students expressed statistically lower perceived levels of social inclusion and emotional inclusion and had a weaker academic self-concept. For example, the mean of emotional inclusion for SEN students was 2.89 (SD =0.975) compared to the mean of 3.47 (SD =0.624) for other students. Based on gender, the mean for girls was higher on this subscale about emotional inclusion; M = 3.49 (SD = 0.63) in comparison with M = 3.24 (SD = 0.76) for boys.

The mean of social inclusion for SEN students was 3.06 (SD =0.905) compared to the mean of 3.44 (SD =0.624) for other students. While the mean for girls on this subscale was M = 3.41 (SD = 0.62) for girls in comparison with M = 3.39 (SD = 0.62) for boys.

And the mean of academic self-concept for SEN students was 2.70 (SD =0.847) compared to the mean of 3.37 (SD =0.585) for other students (see Table 2). The mean for girls on the academic self-concept subscale was M = 3.434 (SD = 0.65) for girls in comparison with M = 3.22 (SD = 0.63) for boys.

**Table 2:** Differences in the perceived levels of inclusion based on whether students have SEN.

Student with SEN	Yes *N* (82)	No *N* (742)	Tests of between-subject effects	*d*
	M (SD)	M (SD)		
EI	2.89 (0.975)	3.47 (0.624)	*F* 1,822 = 55.44, *p* < 0.01	0.74
SI	3.06 (0.905)	3.44 (0.572)	*F* 1,822 = 28.35, *P* < 0.01	0.50
AC	2.70 (0.847)	3.37 (0.585)	*F* 1,822 = 86.49, *P* < 0.01	0.92

EI, emotional inclusion; SI, social inclusion; AC, academic self-concept, and *d*, Cohen’s *d* (effect size).

### Predictors of Emotional Inclusion

To examine whether there were differences based on students’ characteristics (gender, having SEN, ITPS ratings, educational setting on class and school level) in students’ ratings of their perception of inclusion. Three multiple regression analyses were conducted to find out which independent variables would predict students’ perceived levels of emotional inclusion and social inclusion and their academic self-concept. Six predictors were entered at the same time: gender, age, SEN, ITPS, school, and class (Table 3). Four predictors were found to be significant: SEN, age, ITPS, and school. “Students” ratings of inclusive practices’ formed the strongest predictor, with partial *R*^2^ = 0.384. The second strongest predictor was “whether students had SEN,” with partial R = 0.207. The results also showed that the second significant predictor was “whether the student had SEN.” The amount of variation, in terms of emotion, that could be explained by this model with six predictors was around 25.1% (*R*^2^ = 0.251).

**Table 3:** Multiple regression statistics for examining predictors of students’ perceived emotional inclusion levels.

Model		Unstandardized coefficients		Standardized coefficients	*t*	Sig.	Partial *R*
		*B*	Std. error	Beta			
1	(Constant)	0.955	0.273		3.499	0.000	
	ITPS	0.495	0.042	0.375	11.869	**0.000** ^*^	0.384
	Age	−0.054	0.013	−0.140	−4.052	**0.000** ^*^	−0.141
	Class	−0.00010	0.000	−0.028	−0.846	0.398	−0.030
	SEN	0.428	0.071	0.187	6.046	**0.000** ^*^	0.207
	Gender	0.243	0.082	0.163	2.955	**0.003** ^*^	0.103
	School	0.003	0.002	0.102	1.729	0.084	0.061

Bold, significant at *p* < 0.05.

* Significant at *p* < 0.01.

### Predictors of Social Inclusion

In order to examine to examine predictors that might influence students perception of social inclusion, the same six predictors (those found in the previous analysis) were entered into multiple regressions at the same time. Five predictors were found to be significant: SEN, ITPS, gender, classroom, and school. Students’ ratings of inclusive practices formed the strongest predictor, with partial *R*^2^ = 0.433. The second strongest indicator was “whether the students had SEN,” with partial *R* = 0.186. The amount of variation in emotion that can be explained by this model with six predictors is around 21.3% (*R*^2^ = 0.213). For further details, see Table 4.

**Table 4:** Multiple regression statistics for examining the predictors of students’ perceived social inclusion levels.

Model		Unstandardized coefficients		Standardized coefficients	*t*	Sig.	Partial *R*
		*B*	Std. error	Beta			
1	(Constant)	1.547	0.251		6.156	0.000	
	ITPS	0.510	0.038	0.430	13.280	**0.000** ^*^	0.433
	Age	0.019	0.012	0.055	1.549	0.122	−0.021
	Class	0.000	0.000	0.069	2.067	**0.039**	0.077
	SEN	0.225	0.065	0.109	3.447	**0.001** ^*^	0.186
	Gender	−0.213	0.076	−0.159	−2.805	**0.005** ^*^	0.009
	School	−0.004	0.001	−0.160	−2.635	**0.009** ^*^	−0.021

Bold, significant at *p* < 0.05.

* Significant at *p* < 0.01.

### Predictors of Academic Self-Concept

Further, this study carried out a third multiple regression to predict students’ academic self-concept. The same six predictors (those used in the previous analysis) were entered at the same time. Two predictors were found to be significant: ITPS and SEN. Students’ ratings of inclusive practices formed the strongest predictor, with partial *R*^2^ = 0.389. The second strongest indicator was whether the student had SEN, with partial *R* = 0.266. The amount of variation in emotion that could be explained by this model with six predictors was around 24.9% (*R*^2^ = 0.249). For further details see Table 5. In sum, from all three multiple regression (Tables 3–5) we found that high levels of inclusive teaching practices strongly predicted students’ perceived emotional inclusion, social inclusion, and academic self-concept.

**Table 5:** Multiple regression statistics for examining the predictors of students’ perceived academic self-concept.

Model		Unstandardized coefficients		Standardized coefficients	*t*	Sig.	Partial *R*
		*B*	Std. error	Beta			
1	(Constant)	0.745	0.257		2.900	0.004	
	ITPS	0.473	0.039	0.381	12.052	**0.000** ^*^	0.389
	Age	−0.024	0.013	−0.065	−1.885	0.060	−0.066
	Class	−0.00001	0.000	−0.006	−0.170	0.865	−0.006
	SEN	0.524	0.067	0.243	7.857	**0.000** ^*^	0.266
	Gender	0.079	0.077	0.056	1.014	0.311	0.036
	School	0.001	0.001	0.052	0.876	0.381	0.031

Bold, significant at *p* < 0.05.

* Significant at *p* < 0.01.

## Discussion

The importance of students’ educational inclusion is internationally acknowledged and discussed in discourses on scientific and practical levels of education. In the context of inclusive education in particular, the success of students’ social (social participation) and emotional (school wellbeing) development must be addressed (alongside their academic achievement), and the current implementation and practices of inclusive education (e.g., more differentiation and personalization of teaching practices) must be evaluated. In this context, the study investigated Saudi Arabian students’ social inclusion and school wellbeing along with their academic self-concept.

At the beginning, this study examined the psychometric properties of the student version of the Arabic language PIQ. The suggested three-dimensional factor structure was confirmed using CFA. However, the negative items were not perceived correctly by the participating Saudi Arabian students (see Alnahdi and Schwab, 2020 for more information as regard potential language and cultural factors influencing how students responded to the items). Similarly, the results for the reliability of the PIQ subscales also indicated that the negatively worded items caused problems in psychometric quality. After removing the negative items for the subscale “emotional inclusion,” the Cronbach’s alpha reached an acceptable reliability coefficient, while the reliability for the other two subscales remained lower value than 0.7. Generally, the psychometric properties of PIQ (low reliabilities) limited the interpretations of the study and the comparison of the results with other studies using the PIQ (e.g., DeVries et al., 2018; Schwab et al., 2022). In particular, the low psychometric qualities for academic self-concept should be addressed in future research. It should be noted that this study underlines the importance of checking the psychometric qualities of a newly translated scale, even if it has shown good psychometric properties in other language versions (e.g., Venetz et al., 2015).

Answering the first research question regarding the group differences between students with and without SEN, the analysis results indicated for all three dimensions that students with SEN scored lower levels of inclusion compared to their peers without SEN. In particular, a medium-to-large group difference was found for academic self-concept. Generally, previous studies also indicated that SEN students in regular education had lower academic self-concept in comparison with their non-SEN peers (DeVries et al., 2018; Zurbriggen et al., 2018; Alnahdi and Schwab, 2020; Guillemot and Hessels, 2021; Knickenberg et al., 2021). Against this background, Hypothesis 1a as well as Hypothesis 1b can be confirmed in the context of the current study. From a pedagogical perspective, this is worrying, as students’ academic self-concept directly influences their actual academic development (self-enhancement model). However, due to the fact that students’ academic achievement has an impact on their academic self-concept (skill-development model; Guay et al., 2003), it is not surprising that students with SEN had significantly lower scores with respect to this subscale. Confirming the findings of other studies (McCoy and Banks, 2012; Skrzypiec et al., 2016), a further result of the study was that Saudi Arabian students diagnosed as having SEN showed lower levels of school wellbeing in comparison with their peers without SEN. With keeping in mind that the access for students with SEN to regular schools for students without SEN was conducted by installing special classes in the regular school building, but not by opening up regular classrooms for students with SEN and making them fully inclusive, this result seems not surprising either. This educational approach seems more like seeing students with SEN as being tolerated at regular schools but not being included as equivalent students like their peers without SEN. Lastly, the study underpins SEN students’ higher risk for low social participation in the context of educational processes. This result also corresponds to findings reported in previous studies (Avramidis et al., 2018). In conclusion, the Saudi Arabian students with SEN who are enrolled in regular education face the risk of lower social (social participation) and emotional (school wellbeing) development; thus, creating a strong need for improvement of their educational environment.

To date, little evidence has been found to associate students’ socio-emotional variables with inclusive teaching practices. Therefore, this study offers empirical evidence that didactic decisions of teachers (e.g., regarding use of methods, materials and teaching strategies) can influence students’ outcomes beyond academic achievement. For all three dimensions of inclusion following the PIQ, a relation with inclusive teaching practices was found. Therefore, the results regarding whether students’ perception of their teachers’ implementation of inclusive teaching practices predict students’ perception of inclusion confirm Hypothesis 3 of the current study. When students perceived their teachers’ didactic approaches as more inclusive, students’ perception of school wellbeing and social inclusion moved in a more positive direction, along with their academic self-concept. In addition, older students as well as female students experienced higher school wellbeing in comparison with younger students or male students. In terms of social inclusion, while a similar gender effect was observed (although girls felt more socially included,), there was a contrary age effect as younger students perceived a higher level of social inclusion compared to older students.

## Limitations

Although this study presents opportunities for further applications, it is not free of limitations. First, this study’s sample of SEN students was rather small, and thus, no differences between the different types of SENs could be analyzed. Second, it was unclear whether the instrument would allow for direct comparisons of mean scores between students with and without SEN in the Saudi sample. However, in the case of Swiss students (Knickenberg et al., 2021) and German students (DeVries et al., 2018), mean comparisons were allowed. Future studies could verify whether there is a measurement variance between the two groups of students.

## Implications and Future Research

Several conclusions can be drawn from this study. First, this study offers empirical evidence to confirm the importance that inclusive practices on students’ outcomes. For all three dimensions (academic self-concept, social participation, school wellbeing) of inclusive education, a correlation with inclusive teaching practices was supported. Second, the need for more services to help students with SEN who are enrolled in regular education as they face the risk of lower socio-emotional development. Therefore, there is a clear need for improvement in their educational services. For future studies, a replication study with samples from other regions in Saudi Arabia or other Arabic country, especially rural areas, would be recommended. This will allow to examine the level of educational services across different types of regions and to examine whether it vary significantly from region to other. In addition, it would informative for a future study, to examine the impact of level of resources available to inclusive schools on students’ outcomes.

## Data Availability Statement

The raw data supporting the conclusions of this article will be made available by the authors, without undue reservation.

## Ethics Statement

Approval for this study was obtained from the institutional review board (IRB) of Prince Sattam Bin Abdulaziz University. Written informed consent to participate in this study was provided by the participants’ legal guardian/next of kin.

## Author Contributions

All authors listed have made a substantial, direct, and intellectual contribution to the work and approved it for publication.

## Funding

This work was supported by Deanship of Scientific Research at Prince Sattam Bin Abdulaziz University (RG #2021/02/18510).

## Conflict of Interest

The authors declare that the research was conducted in the absence of any commercial or financial relationships that could be construed as a potential conflict of interest.

The handling editor MN and the reviewer MN declared a shared affiliation, with no other collaboration, with the author SS at the time of review.

## Publisher’s Note

All claims expressed in this article are solely those of the authors and do not necessarily represent those of their affiliated organizations, or those of the publisher, the editors and the reviewers. Any product that may be evaluated in this article, or claim that may be made by its manufacturer, is not guaranteed or endorsed by the publisher.
